# Progress Report: Antimicrobial Drug Discovery in the Resistance Era

**DOI:** 10.3390/ph15040413

**Published:** 2022-03-28

**Authors:** Pottathil Shinu, Abdulaziz K. Al Mouslem, Anroop B. Nair, Katharigatta N. Venugopala, Mahesh Attimarad, Varsha A. Singh, Sreeharsha Nagaraja, Ghallab Alotaibi, Pran Kishore Deb

**Affiliations:** 1 Department of Biomedical Sciences, College of Clinical Pharmacy, King Faisal University, Al-Ahsa 31982, Saudi Arabia; 2 Department of Pharmaceutical Sciences, College of Clinical Pharmacy, King Faisal University, Al-Ahsa 31982, Saudi Arabia; aalmoslem@kfu.edu.sa (A.K.A.M.); anair@kfu.edu.sa (A.B.N.); kvenugopala@kfu.edu.sa (K.N.V.); mattimarad@kfu.edu.sa (M.A.); singhvarul@gmail.com (S.N.); 3 Department of Biotechnology and Food Science, Faculty of Applied Sciences, Durban University of Technology, Durban 4000, South Africa; 4 Department of Microbiology, Rajmata Vijaya Raje Scindia Medical College, Rajasthan University of Health Sciences, Jaipur 302033, India; sharsha@kfu.edu.sa; 5 Department of Pharmaceutical Chemistry, Vidya Siri College of Pharmacy, Off Sarjapura Road, Bangalore 560035, India; 6 Department of Pharmaceutical Sciences, College of Pharmacy, Shaqra University, Riyadh 11961, Saudi Arabia; ghalotaibi@su.edu.sa; 7 Department of Pharmaceutical Sciences, Faculty of Pharmacy, Philadelphia University, Amman 19392, Jordan; prankishore1@gmail.com

**Keywords:** antimicrobial resistance, drug discovery, drug screening, antimicrobial targets, MDR, adjuvants, drug targets

## Abstract

Antibiotic resistance continues to be a most serious threat to public health. This situation demands that the scientific community increase their efforts for the discovery of alternative strategies to circumvent the problems associated with conventional small molecule therapeutics. The Global Antimicrobial Resistance and Use Surveillance System (GLASS) Report (published in June 2021) discloses the rapidly increasing number of bacterial infections that are mainly caused by antimicrobial-resistant bacteria. These concerns have initiated various government agencies and other organizations to educate the public regarding the appropriate use of antibiotics. This review discusses a brief highlight on the timeline of antimicrobial drug discovery with a special emphasis on the historical development of antimicrobial resistance. In addition, new antimicrobial targets and approaches, recent developments in drug screening, design, and delivery were covered. This review also discusses the emergence and roles of various antibiotic adjuvants and combination therapies while shedding light on current challenges and future perspectives. Overall, the emergence of resistant microbial strains has challenged drug discovery but their efforts to develop alternative technologies such as nanomaterials seem to be promising for the future.

## 1. Introduction

**Figure 1:**
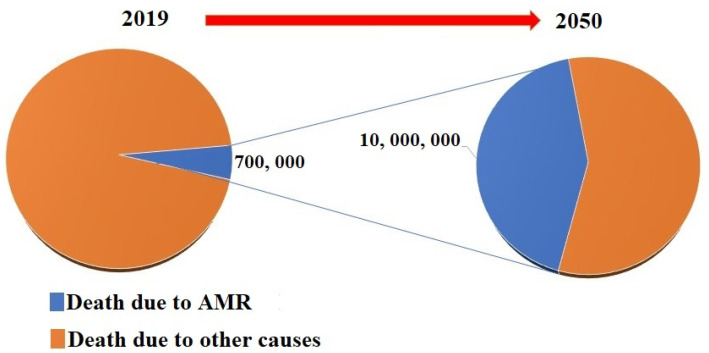
Pie chart showing current number of antimicrobial-resistant mediated global deaths and expected number of global deaths due to antimicrobial-resistant infectious diseases in 2050.

Antibiotic resistance stands as one of the most serious threats to public health. In 2019, the Center for Disease Control and Prevention (CDC) published a report which shows that >2.8 million individuals were infected with antibiotic-resistant bacteria in the US [1]. These antimicrobial-resistant infections further contribute to the death of >35,000 people every year [1]. A research report published by the World Health Organization (WHO) revealed that global deaths directly caused by antimicrobial-resistant pathogens are expected to number 10 million per year by 2050, as shown in Figure 1 [2,3]. This global threat has encouraged the scientific community to increase their efforts to research and discover alternative strategies to circumvent the problems associated with conventional small molecule therapeutics [4].

As the epidemiological transition hit, pandemics and infectious diseases have started to recede, causing a significant increase in people’s life expectancy around the globe. As a result, the disease burden structure has moved to cover civilizational illnesses, rendering cancer, cardiovascular diseases, and degenerative diseases the main leading causes of death [5]. Improving sanitation, introducing antibiotics and vaccines as well as the overall development of public health are among the most significant reasons for the reduced morbidity and mortality of infectious diseases [6]. The discovery of antibiotics and their clinical use have played a vital role in medicine, resulting in a revolution in care for patients who have been infected with common or deadly bacterial agents that were affecting the respiratory tract, urinary tract, gastro-intestinal tracts, and skin and soft tissues [7]. The discovery of antibiotics has saved millions of lives since the 1950s and paved the way for developing complex medical specialties and interventions, something which was not an achievable goal in early times [8]. In COVID-19 units, sporadic antibiotic-resistant outbreaks have been reported. This may be attributed to the long-term use of broad-spectrum antibiotics during COVID-19 treatment. Indeed, the higher rates of hospital-onset infections in these patients are caused due to pre-existing viral infection-induced sepsis. However, the emergence of antimicrobial resistance is set to be one of the most life-threatening concerns of the millennia [9]. The reasons for the current antimicrobial crisis may be attributed to various factors including the general failure of a careful grasping of this evolution, of the sources and dissemination, and of the molecular mechanisms of antimicrobial resistance. Moreover, the lack of sufficient studies on the chemical and biophysical mechanisms of antimicrobial resistance are proposed to be the main reasons for drug failure [10]. In addition, other factors, such as genetic diversity and potential environmental reservoirs have been shown to fall out of consideration in the process of drug development. Therefore, a more integral understanding of origin, diversity, as well as resistance mechanisms could guide the discovery of new drugs, and the development of mitigation strategies against resistance, strategies such as antibiotic adjuvants, the implementation of nanomaterials for enhanced drug delivery, etc. [11,12]. This review article is aimed to highlight the recent progress in the discovery and development of antimicrobial agents and the latest strategies developed to circumvent antimicrobial resistance. New antimicrobial molecules, targets, and approaches have been discussed with a special highlight on the use of nanomaterials, antibiotic adjuvants, and some of the combination strategies under evaluation.

## 2. Selection of Literature

**Figure 2:**
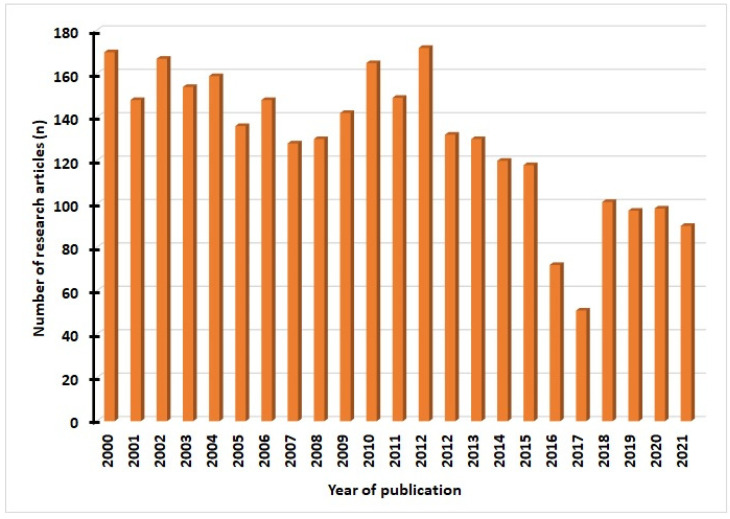
The number of research publications in antimicrobial resistance from 2000 to 2021.

Relevant articles were obtained from Science Direct, Medline, Public Library of Science, Mendeley, PubMed, Springer Link, and Google Scholar. We used various keywords such as “antimicrobial resistance”, “adjuvants”,” nanodots”, “drug targets”, “stimuli-responsive nanocarriers”. We selected articles in the English language from 2000 to 2021 and analyzed them in this review article. Of the total articles (*n* = 2984) obtained, we excluded irrelevant manuscripts after analyzing titles and abstracts but included those that were potentially relevant (*n* = 165) for the final analysis. The details of articles related to this work published per year are shown in Figure 2.

## 3. Antimicrobial Drug Discovery: Timeline

Screening of natural products was known historically as the most successful approach to discovering antibacterial agents. To date, the majority of the antibiotics available in the market were either natural compounds, or semi-synthetic or synthetic compounds that were obtained from natural sources [13]. There are several approaches to be employed to discover natural antimicrobials, and they can be classified into diversity-based approaches, such as culturing the “uncultured” bacteria and co-culture, and approaches such as ribosome engineering, genome mining, and genetic engineering [14]. The potential of nanomaterials technology and mineral-based technology against microbe infections have been extensively reviewed in recent literature [15,16,17]. 

Recent advances in combinatorial chemistry and computational models have given support to the rational discovery of new agents and improvement of the activity of the antimicrobials that currently exist [18,19]. It is evident that less than 1% of the microbes in the environment are capable of being cultivated in the laboratory [20]. Thus, nutrient media, as well as diffusion chambers, have been applied in high-throughput cultivation for a number of microbes (e.g., marine isolates) to support the global effort in cultivating new microbial taxa. Soil Actinomycetes, and Streptomyces species, in particular, have been the main source that was used to develop most of the natural antibiotics available nowadays [21]. Following decades of the exploitation of terrestrial Streptomycetes, researchers have started to investigate other sporadic Actinomycetes and bacteria taxa including Cyanobacteria and Proteobacteria [22]. Consequently, a number of novel Actinomycetes species along with the various natural products acting on these species have been discovered in hyper-arid desert soils, deep ocean sediments, as well as hot springs to designate a few unfamiliar habitats [23]. In addition, Endophytic bacteria [24] living in rhizospheres and plant tissues, symbiotic bacteria including the actinobacteria bacterial-nematode associations [25], and human commensals have been recently employed as sources to discover various novel antibiotics [4]. In a study, a bioprospecting strategy was outlined in which species living in extreme or uncommon habitats were cultivated using certain isolation methods, which were then followed by screening for natural products [26]. It is rare for micro-organisms to be found isolated in the environment and reports have revealed that micro-organisms usually grow in co-culture with other species. This co-culture’s existence was shown to induce antibiotic production [27]. Moreover, co-cultures were shown to lead to an induction of silent antibiotic genes as a result of competition [4,14,28]. Since the mid-1960s, challenges concerning the use of the Waksman platform approach to identify novel and effective antibiotic scaffolds arose [29,30]. Consequently, new scientific and commercial challenges are creating a huge gap between the clinical need to develop new antimicrobial agents in the resistance era and new approaches in drug discovery and development [31,32,33]. These challenges and the associated low output-to-input ratio have led pharmaceutical industries in the past two decades to adopt new programs to run the different stages of antibiotic discovery while shedding expertise in antimicrobial drug development [34]. Therefore, only a few new antibiotic classes such as daptomycin [35] have been approved by the Food and Drug Administration (FDA) recently [2]. Despite the initiatives proposed by governments, non-profits as well as public health organizations by providing arrangements (for instance; increased investment) to provoke enthusiasm for the development of novel antibiotics, the success of these schemes has been restricted. In addition, anti-virulence agents, probiotics, antibodies, as well as vaccines are other alternatives that have been recently investigated as potential alternative therapies [36,37]. However, they were still shown to act best as clinical preventive or adjunctive therapies due to their unguaranteed effectiveness and their safety for use as monotherapies, something which makes the use of conventional antibiotic therapies indispensable. Accordingly, developing combination therapies has shown to be a promising avenue to avoid investment in the development of new drugs [38]. In particular, repurposing antibiotic adjuvants that have already undergone wide pharmacological and toxicological analysis is thought to be an effective approach that would cause a reduction in the time, risks, and costs associated with the traditional antimicrobial innovation [2,39,40]. The various timeline-dependent strategies used in drug discovery are listed in Table 1 [41].

## 4. Historical Aspects for the Development of Antimicrobial Resistance

**Figure 3:**
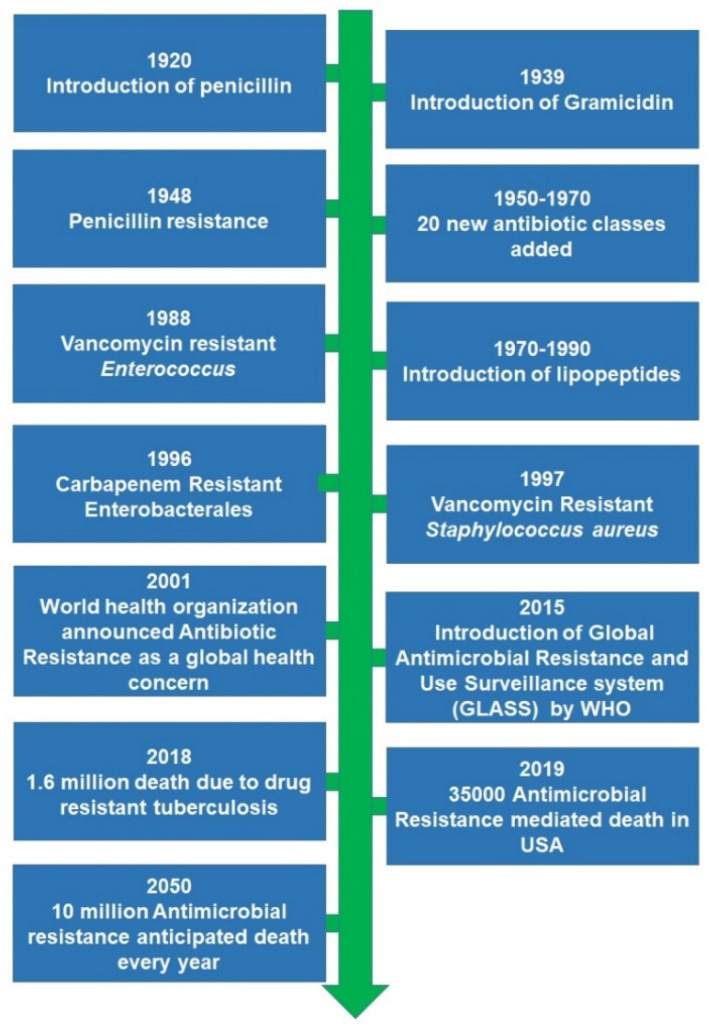
Schematic representation of historical aspects for the development of antimicrobial resistance.

The first incident reported as an antimicrobial therapy’s failure in humans as a result of acquired microbial resistance goes back to the 1940s for penicillin (**1**), only a few years after it was commercialized [42]. The revolution in the therapeutics discovery that was initiated by penicillin paved the way to discover various approaches. Gramicidin (**2**) was reported as the first antimicrobial peptide (AMP) discovered in 1939 after being obtained through isolation from Bacillus brevis. However, Defensin has been reported as the first animal antimicrobial peptide reported in the literature after its isolation from rabbit leukocytes [43]. After the 1960s, due to the noticeable increase in MDR microbial pathogens, global interest and research efforts were dedicated to antimicrobial peptides, with over 5000 AMP being documented [44]. The evolution of acquired microbial resistance to several marketed drugs has been growing over the 20th and early 21st centuries [44,45]. Out of the most life-threatening drug-resistant infections, extensively drug-resistant (XDR) and multidrug-resistant (MDR) strains of tubercle bacilli, carbapenemase-producing Enterobacterales (CRE), and vancomycin-resistant *Staphylococcus aureus* (VRSA), as well as some antimicrobial-resistant strains of *Clostridiodes difficile* have accounted for serious health concerns that require a special focus in research [46,47,48]. In addition, *Enterococcus faecium, Staphylococcus aureus*, *Klebsiella pneumoniae*, *Acinetobacter baumannii, Pseudomonas aeruginosa*, and *Enterobacter species*, also known as ESKAPE, has recently emerged with the ability to withstand lethal doses of almost all antibiotics. In a recent report that has been published by the World Health Organization (WHO). It has been estimated that acquired antimicrobial resistance (AMR) is anticipated to be the leading cause of death for 10 million people every year by 2050 [49]. A schematic representation of historical aspects for the development of antimicrobial resistance is shown in Figure 3.

Various international health agencies such as the W.H.O., the British Society for Antimicrobial Therapy, the National Institute of Health, and the Centre for Disease Prevention and Control (CDC) have reported on the current global antimicrobial crisis [50,51]. This serious antimicrobial crisis may be attributed to various factors such as a lack of availability of novel antimicrobial agents, lack of appropriate strategy for the discovery of new antibiotics, the ineffectiveness of pre-existing antibiotics for treatment, the high rate of mutation occurring among common pathogens, abuse, and lack of awareness about the proper use of antibiotics [4,52].

A recent study that has been conducted and published in the USA reports that a minimum of two million people have been infected annually with drug-resistant strains of bacteria. This has further caused the death of 23,000 patients due to antibiotic treatment failure. However, the percentage reported in developing countries such as India has been shown to be 2–3 times higher [53,54,55,56]. 

In a report that has been published by the center for disease control and prevention (CDC) in 2019, it is reported that about three million antibiotic-resistant infections occur annually in the USA, leading to about 35,000 deaths. Additionally, 223,900 cases of *Clostridioides difficile* have also been observed, causing the death of 12,800 patients [57]. In another recent report that has been published by the WHO, the top 10 globally threatening health issues in 2019 are revealed, showing AMR to tuberculosis drugs accounting for a huge contribution with about 1.6 million annual deaths in 10 million cases [41,58,59,60,61].

The WHO has also published different documents as a way of guiding the development of the new antimicrobial, such as the published list of global priority pathogens and the resultant target diseases [49]. In 2015, the WHO introduced the Global Antimicrobial Resistance and Use Surveillance System (GLASS), which is the first system adopted globally for collecting official national data about antimicrobial resistance, in particular bacterial pathogens [62]. Recently, the GLASS report of June 2021 has shown that the number of antibiotic-resistant bacterial infections particularly against currently available antibiotics on the market is increasing drastically [62]. Moreover, concerns have started to raise regarding the lack of awareness towards the appropriate use of antibiotics, especially at the time of the COVID- 19 pandemic, which have made the WHO issue instructions to avoid recommending antibiotics or prophylaxes for patients who have mild to moderate COVID-19 except if a bacterial infection’s signs and symptoms exist [4,63,64]. 

## 5. Antimicrobial Peptides as New Molecules against Drug Resistance

Antimicrobial peptides (AMPs) are short peptides with a large distribution in nature. Since their discovery, about 3197 antimicrobial peptides have been placed in the Antimicrobial Peptide Database record. It has been reported that about 2374 peptides were derived from animals, 352 were obtained from plants, 356 from bacteria, 20 from fungi, and finally, 13 from archaea and protists [65]. Moreover, the data show that 83.7% of AMPs were shown to exhibit broad-spectrum activity which means that they can act on gram-negative and positive bacteria. Some examples of AMPs are magainin 2 (**3**) [66,67], indolicidin (**4**) [68,69], protegrin (**5**) [70,71], and human β-defensin-3 [72]. A percentage of 36.1% was reported with anti-fungal activity, as was reported in a study that showed that Dermaseptin had actively killed yeast *Candida albicans* [65,73,74]. AMPs were found to have residues of hydrophobic and cationic amino acid which, upon absorbing into the bacterial membrane, can undergo segregation into cationic or hydrophobic patches, giving these peptides the membrane-active antimicrobial activity. Natural AMPs that are proposed for clinical use are restricted by some challenges, as they are toxic, have low-to-moderate efficacy in vivo, are expensive in terms of development and production, and have low stability towards protease enzymes. Nonetheless, synthetic approaches have been adopted for optimizing the efficacy, safety as well as stability of AMPs for a further understanding of their structure–activity relationship (SAR) [65,75]. In terms of protease instability, it has been suggested in a study that replacing the proteinogenic L-amino acids with D-amino acids would render the peptide more protease-resistant. Merrifield et al. have conducted a study where they replaced the L-amino acids with D-amino acids in magainin 2 and melittin (**6**). Results have shown that all the D-enantiomers exhibited similar antimicrobial activities as compared with their natural counterparts with less toxicity and a higher resistance towards protease degradation [76,77,78,79]. It is worth mentioning that replacing some AMPs amino acid residues do not necessarily lead to antimicrobial activity loss, however, sometimes it may enhance it, which shows that a specific type or sequence of amino acids is not a necessity for acquiring the antimicrobial activity and that the overall physiochemical properties are the main factors determining AMPs. In summary, synthetic peptides and some peptide mimics are reported to have higher stability, better activity, and selectivity [80]. However, due to the requirement of a stepwise synthesis of these agents, more effort and time are still needed. Currently, the majority of AMPs and SMAMPs are synthesized to adopt a fatty acid (FA) structure [65]. Table 2 summarizes the currently available AMPs databases [81]. Extensive clinical investigations of several AMPs and their future potential for their commercialization are described in a recent review [82]. 

## 6. New Antimicrobial Drug Targets

### 6.1. NagZ Protein

NagZ is a cytosolic glucosaminidase that is found to play a role in the recycling of peptidoglycan as it is able of hydrolyzing N-acetylglucosaminyl-1,6-anhydromuropeptides (peptidoglycan monomers) to form N-acetylglucosaminyl (GlcNAc) and 1,6-anhydromuropeptides (anhMurNAc) [83]. In a study that has been carried out recently, results have revealed that inactivating NagZ has been shown to inhibit and reverse 𝛽-lactam resistance in *P. aeruginosa* [84], *Y. enterocolitica* [85], as well as *Stenotrophomonas maltophilia* [86]. Moreover, NagZ has also been shown to have a moonlighting activity that is responsible for modulating the accumulation of biofilms in Neisseria gonorrhoeae [87]. These promising findings have encouraged researchers to study this new target more thoroughly, however, the precise regulation of this protein to resistance is still unknown in *Enterobacter cloacae (EC)*. A recent study has been carried out to ascertain the mechanism of NagZ in the development of resistance in EC and the regulation of chromosomal mediated AmpC 𝛽-lactamase production. It is evident from reports that NagZ has been overexpressing in cephalosporin-resistant *Enterobacter cloacae* when compared with in cephalosporin susceptible *Enterobacter cloacae*. This suggests that complementation of NagZ enhances EC cephalosporin resistance by up regulating the Amp-C expression. It is found that NagZ hydrolyzates 1,6-anhydromuropeptides (anhMurNAc), which have also been shown to prompt the expression of AmpR genes [88]. Other studies have suggested over the last decade that blocking the recycling process of peptidoglycan might attenuate the AmpC-mediated resistance in *P. aeruginosa*, and some inhibitors have been developed and tested such as PUGNAc, MM-124, EtBuPUG, and MM-156 [89]. However, in vivo validation of the targeting of peptidoglycans recycling by antibiotic adjuvants is still missing. A study has shown that inactivating NagZ protein, and particularly the AmpG, has increased the in vivo treatments’ effectiveness, and hence reverted 𝛽-lactam resistance [90]. However, it should be noted that in vitro studies have shown that this effect is detectable in AmpC-hyperproducing strains only as demonstrated in other studies. Nevertheless, some studies have concluded that using the peptidoglycan recycling pathway as a co-target in some combination therapies can also be beneficial [41,91,92]. 

### 6.2. AmpG Protein

The ampG protein has a role in encoding the transmembrane permease in various gram-negative bacteria such as *Pseudomonas aeruginosa*, *Acinetobacter baumannii*, *Vibrio cholerae*, and *E. coli* [93]. AmpG permease is the enzyme responsible for transporting fragments of peptidoglycan such as 1, 6-GlcNAc-anhydro-MurNAc, and 1, 6-GlcNAc-anhydro-MurNAc peptides, which are required for pepridoglycan recycling in bacteria. AmpG permease was also shown to contribute to the 𝛽-lactamase induction pathway [94]. Studies have revealed that inhibiting the AmpG protein will deplete beta-lactamase enzyme, which therefore suggests that designing AmpG inhibitors such as fosfomycin (**7**) might be beneficial in suppressing the 𝛽-lactamase release in microbes, and that these agents might be more suitable as adjuvants to the conventional 𝛽-lactam antibiotics [95]. Other studies have also shown that inhibiting AmpG protein will terminate the process of cell wall recycling in bacteria; which significantly enhances the wall’s permeability, suggesting that these agents can be used as permeability enhancers to all antibiotics exhibiting high potency (i.e., fluoroquinolones) [96]. It is worth mentioning that the AmpG protein acts as a target and may be more suitable among the other targets. This is mainly due to the proven 𝛽-lactamase expression mechanism of AmpG that has been reported in drug-resistant bacteria as well as being more reliable on the basis of the site of action, since it acts on the cell membrane as compared with NagZ which acts on the cytoplasm instead [41]. In support of the targeting of the AmpG protein, a study has shown that interfering with the peptidoglycan recycling has been shown to reduce virulence in animal models, with a significant reduction in virulence in AmpG mutants of *P. aeruginosa* as compared to the parental strain in murine infection models. Another study has shown that interfering with peptidoglycan recycling has shown a reduction in the systemic infection mortality in mice as well as having lower bacterial loads in the lung. Borisova and his colleagues reported in a recent study that the recycling pathway of *P. aeruginosa* and other Gram-negative bacteria are involved in the UDP-MurNAc pool, hence bypassing its de novo synthesis and leading to an intrinsic resistance to the MurA-targeting antibiotic fosfomycin [90,91]. Other studies have shed light on the significant effect of AmpG protein on the *E. coli* ability to form biofilms since there has been an extreme reduction of about 74% in the biofilm formation in its absence. This reduction has also been observed in the presence of 𝛽-lactamases. Molecular genetics studies have shown that deleting AmpG permease would cause a dramatic increase in the 𝛽-lactam susceptibility in presence of AmpC, TEM-1, and OXA beta-lactamases. In addition, this permease, when expressed in excess, will compromise the bacterial cells’ integrity, which would eventually lead to cell lysis. Thus, it could be concluded as stated in a recent study that AmpG permease can act as a prospective antibiotic target which, upon inhibition, could stop both 𝛽-lactamase inductions as well as biofilm formation [94]. In a recent study, an ELQ prodrug (ELQ-502) that shows a high potency and selectivity has been identified as efficient, safe, having excellent bioavailability and a long half-life, and can be used in vitro (i.e., mouse model of parasitemia) either alone or in combination with atovaquone (**8**) to eliminate *B. microti* and *B. duncani* infections. The promising characteristics of this experimental therapy suggest that it would be ideal as a clinical candidate to treat Babesia and apicomplexan parasites’ human infections. In the same study, the site-directed mutation has been performed for two conserved amino acids (I53 and W59), which showed a drastic reduction in the AmpC activity of the mutated amino acids in the transmembrane regions, suggesting that the conserved amino acids have a significant role in preserving the normal functioning of AmpG [97]. 

### 6.3. Polyphosphate Kinase

Inorganic polyphosphates (polyP) are a type of linear polymer, composed of residues of orthophosphate (Pi), that are linked through phosphoanhydride bonds of high energy. The polyP is found in many bacterial species such as Helicobacter pylori. Cytoplasmic polyP is easily spotted as particles tightly compacted at the flagellar pole. PolyP has also been shown to play critical roles in motility, biofilm formation, resistance to quorum sensing, heat, osmotic, oxidative as well as alkaline stresses, and stationary-phase survival [98]. There are three main polyphosphate kinase enzymes, namely, PPK1, PPK2, and PPK3 that have been reported to be responsible for the inorganic polyphosphate accumulation in microbes through animal models [41,99]. A recent study has been conducted to develop novel inhibitors to act against the *E. coli* PPK1 enzyme by adopting in silico and in vitro methods. In the same study, three-dimensional pharmacophores for PPK1 inhibitors have been generated utilizing structure-based assessment of the PPK1 active site. A further investigation of the potential inhibitors has been performed using the biofilm formation assay and phenotypic microarray technique [100,101]. Among all the documented polyphosphate kinase enzymes, the role of PPK2 in developing drug resistance, virulence as well as enhancing cell wall permeability to polar TB drugs have been well studied. Recent studies have revealed that the use of PPK2 inhibitors has shown an increase (8 fold) in the anti-tubercular activity of isoniazid and other drugs of high polarity [41]. Thus, it is beneficial to design and develop new PPK2 inhibitors (e.g., NSC 35676 (**9**), NSC 30205 (**10**), and NSC 345647 (**11**)) to serve as adjuvants to the existing antitubercular drugs and other antibiotics [102]. Bashatwah et al., have reported in a study where the 3D pharmacophores for a number of potential inhibitors to act on a PPK1 enzyme were generated by the utilization of structure-based models. In the same study, three inhibitors (i.e., Inh1, Inh2, and Inh3), were assessed as PPK1 inhibitors using the same approaches of phenotypic microarray methodology and biofilm formation inhibition as mentioned previously in another study. Results have shown that upon biological testing, Inh1 and Inh2 have shown activity in the micromolar concentration range. Moreover, the chemical inhibition of ppk1 was correspondent to the gene deletion in PPK1, suggesting their ability to suppress the biofilm layer formation and hence their potential to be used to overcome bacterial virulence and stress adaptation [100]. Cloete et al. selected three ligands (i.e., ZINC41125011, ZINC20318248, and ZINC20321877) that previously showed good pharmacokinetic properties and toxicity profiles to carry out molecular dynamic (MD) simulation and molecular generalized born surface area (MM-GBSA) analysis. Results have shown that all the three ligands have been relatively stable at the PPK binding site, suggesting that these ligands can be potential inhibitors of MTB following the positive experimental validation [101,103,104]. 

### 6.4. Cytochrome bc1 Complex

The cytochrome bc1 complex of the prokaryotic energy transducing and the eukaryotic mitochondrial membranes is considered as a potential antimicrobial target as stated in medical and agricultural studies [105,106]. Inhibiting cytochrome bc1 complex has been shown to cause ATP depletion as its main bactericidal mechanism of action [41]. Several studies have focused on studying the quinolones as bc1-targeting antimalarials [107,108]. Thus, a broad set of alkyl- and alkoxy 4(1H)-quinolones were synthesized for determining the structure–activity relationship (SAR) for their efficacy as antimalarial agents that show activity against asexual *P. falciparum* parasites, and atovaquone-resistant lines in particular [109]. This has resulted in the discovery of ELQ-300 (**12**) and P4Q-391 (**13**) which have been introduced by the Medicines for Malaria Venture (MMV) as a preclinical candidate (ELQ-300) and a backup molecule (P4Q-391). However, it should be noted that, despite the desirable antimalarial efficacy of endochin and other alkyl-4(1H) quinolones, their metabolic instability has limited their development as they have a long alkyl chain. A study that has been carried out lately has shown that replacing the long alkyl chain with another side chain of pyridone series has overcome the metabolic instability issue while retaining the antimalarial efficacy [110]. 

Inhibiting the parasite cyt bc1 has been reported to take place at the Qi site due to the QSAR being directed against parasites that show resistance toward atovaquone, which harbors Qo-site mutations. In vitro analyses of ELQ300 and atovaquone have investigated their efficacy when combined in displaying a synergistic interaction and suggested that Qo and Qi inhibitors make a more desirable combination strategy [107]. A study has been conducted recently to evaluate the SAR, efficacy, safety as well as mechanism of action of ELQs using blood-borne pathogen *Babesia duncani*. An ELQ prodrug (ELQ-502) of high potency and selectivity has been identified which has been shown to eliminate *B. microti* and *B. duncani* infections. The reported high efficacy, safety, and bioavailability as well as the long half-life have made this prodrug an ideal candidate for clinical use to treat human infections caused by Babesia and other apicomplexan parasites [111]. The above-mentioned new antimicrobial drug targets NagZ Protein, AmpG Protein, Polyphosphate Kinases (PPK1, PPK2, PPK3), and Cytochrome bc1 Complex are promising for the development of novel drugs with improved potency and safety profiles. However, further detailed studies on these targets would provide better insight into the precise regulation mechanism of these proteins.

## 7. Recent Developments in Drug Discovery against Drug Resistance

Recent reports have revealed that a percentage exceeding 90% of the total hit molecules developed lately were labeled “not suitable” for the treatment of drug-resistant infections, whereas the remaining molecules (including Oritavancin and Dalvance) were shown to either have low potency against super bugs or showing a high level of cytotoxicity for clinical use in humans. These statistics have made the WHO declare that we are currently in the antibiotic crisis era, showing a necessity to investigate new agents while developing enhanced strategies at the same time [41]. As recommended by the WHO, the MDR-TB treatment is initiated with an injection-free therapy (groups A and B). However, in cases where group A and B drugs cannot be given, group C oral and parenteral drugs are given instead. During lengthy treatments and in the cases where there is a lack of patient compliance, mutations in genes encoding drug targets that are responsible for developing resistances in clinical strains occur [112]. A list of the major target genes that confer resistance in cases of mutations is shown in Table 3 [113].

### 7.1. Drug Screening and Non-Conventional Growth Media

In general, there are two main principles for developing effective drug discovery programs. These drug discovery programs include: (i) the target-oriented screening, which aims to facilitate the identification of compounds that have good compatibility with an identified and validated molecular target; and (ii) the bioactive-guided screening, which aims to analyze the molecular target as well as the compound’s mode of action following the active substance’s identification [13,130,131]. Protocols for high-throughput screening as well as de novo design are usually utilized for establishing new drugs along with identifying their antimicrobial structure-activity relationship [4]. Aside from the conventional screening approaches that rely on the growth of bacteria, the innovative technological advancements in both sequencing, as well as random transposon mutagenesis, enhanced the new screening techniques that use novel antibiotic adjuvants (obtained from massive libraries composed of non-antibiotic agents) [13]. An experimental–computational approach that functions on basis of high-throughput metabolomics has been recently developed in a study for the prediction of drug–drug interaction. In the same study, high-throughput metabolomics has been applied for monitoring *E. coli* metabolic response to a library of 1279 chemical agents, where the majority of these compounds were human-targeted and had little or no direct antimicrobial activity. De novo predictions to study and identify the modes of drugs’ action have been made by the authors through a combination that gathered drug metabolome profiles that had been newly generated with fitness and metabolic profiles in *E. coli* gene-knockout mutants, mutants which have systematically shown epistatic drug–drug interactions. Results have shown that numerous novel drug combinations (i.e., sulfamethizole (**31**) and zidovudine (**32**)) were identified in that study for the first time [132]. Nevertheless, exploring the chemical–genetic interactions is another promising approach to identify novel synergistic small-molecule pairs, and the overlap2 method (O2M) is one representative example [133]. In this method, identified synergistic interactions are applied for predicting many supplementary interactions using large-scale chemical-genetic data. Studies have shown that this method has successfully screened synergistic small-molecule pairs to combat antibiotic-resistant bacteria. Consequently, about 2000 small molecules have been screened (e.g., azidothymidine) and were found to have a synergistic effect, as well as acting against resistant clinical *E. coli* and *K. pneumoniae* when combined with trimethoprim and/or sulfamethazine [134]. Additionally, the antibiotic resistance platform (ARP) is a new platform that has been applied by a recent study by which each different resistance element has been cloned into a uniform *E. coli* host [135]. Subsequently, a selection of transformed *E. coli* capable of expressing one of greater than 40 different identified antibiotic resistance genes (ARGs) has been constructed. Moreover, this platform has also been shown to present a streamlined screening and testing tool for improving the conventional drug-resistant pathogens’ screening, which is often known to have redundant resistance elements and poorly-characterized genotypes. However, it should be noted that as the platform is designed by engineered *E. coli* that has a completely-sequenced genetic background, it is unfeasible that one can decide if the screened compounds are going to have equivalent efficacy when acting against clinical drug-resistant strains [2].

### 7.2. Nanomaterials for the Design and Delivery of Antimicrobial Agents

**Figure 4:**
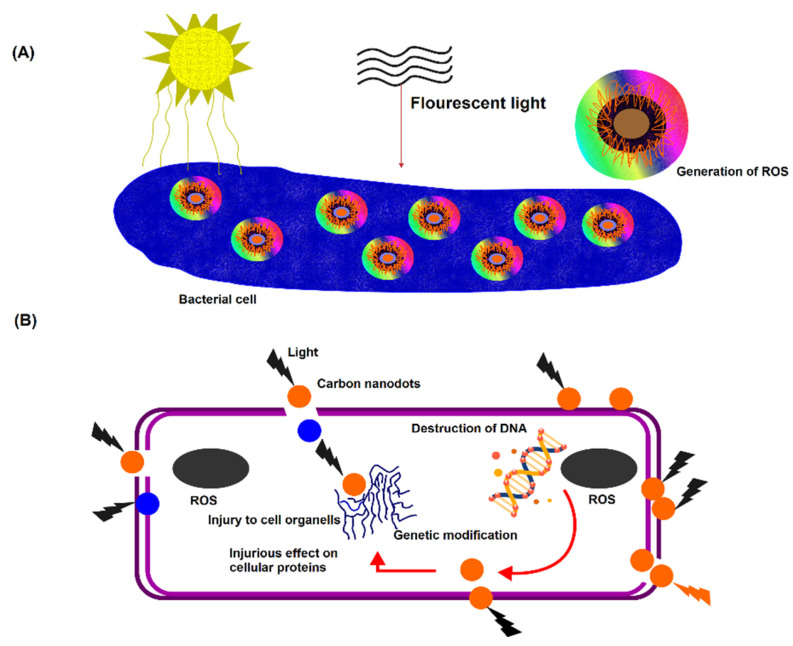
An illustration representing the mode of action of carbon nanodots against bacterial cells. (**A**) Adhesion of carbon nanodots to the bacterial cell surface, and the visible-light-induced ROS generation. (**B**) ROS mediated intracellular bacterial cell damage.

Researchers have progressively begun to consider alternative methodologies for mitigating the global crisis of antimicrobial resistance. A more focused approach has been directed toward preventing the occurrence of antimicrobial resistance, whereby the overall undesirable effects from bacteria showing resistance on public health are believed to be weakened by decreasing the infections rates within the population. Carbon nanodots, also known as quasi-spherical nanoparticles, made from multiple layers of oxidized graphene sheets, are promising alternatives, as shown in Figure 4 [136,137]. Carbon nanodots are a combustion byproduct that are collected in a simple, rapid, and inexpensive way from low-heat flames. Furthermore, these particles are also frequently reported for their resistance against photodegradation and have been reported in a study to be stable for this use, suggesting that they are advantageous assets in terms of shelf-life [138]. Despite being primarily studied for fluorescence applications (i.e., diagnostics) [139], carbon nanodots have recently gained more interest as antimicrobial agents, as shown in some reports where these nanodots’ intrinsic antimicrobial activity [140], and their synergistic toxicity with antibiotics have been investigated [141]. A study has recently demonstrated that these particles’ composition can be adjusted to acquire luminescence characteristics to be used for APDI [142]. It has been reported that incorporating bromine into the carbon nanodots to form a brominated carbon nanodot structure (BrCND) has permitted efficient spin–orbit coupling and hence phosphorescence detection due to a heavy atom effect phenomenon. Furthermore, a recent study conducted by Zhang et al. has linked the carbon dots’ photodynamic antimicrobial activity against *E. coli* and *Salmonella species* to their phosphorescent properties, where they cited nitrogen as the phosphorescence-tuning source in the dots. In the same study, it has been demonstrated that these dots’ structures display a photosensitization efficacy that is competitive to photosensitizers (e.g., phloxine B and rose Bengal) [140,143]. 

**Figure 5:**
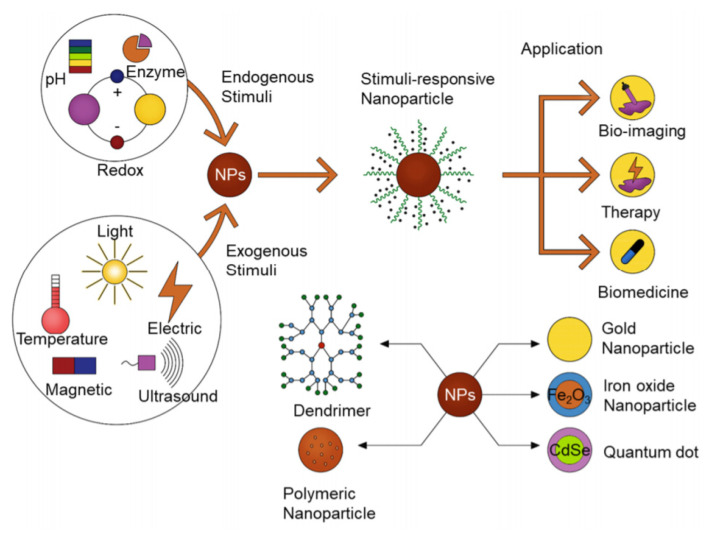
A schematic illustration of stimuli-responsive nanomaterials for different applications including therapy, bio-imaging as well as triggered drug release (Reprinted from ref. [144]).

A significant improvement in the design and development of nanomaterials has become evident worldwide. Intrinsic stimuli-responsive antibiotic nanocarriers are one example of these novel nanomaterials that have been recently developed and shown to have improved activity, enhanced targeted delivery, as well as having a superior potential for bacterial penetration and eradication, as shown in Figure 5 [144]. Stimuli-responsive nanocarriers can specifically enhance drug release and targeting profiles unlike the traditional nanocarriers [145]. Stimuli-responsive nanocarriers are also capable of identifying the bacterial and infection site’s distinct microenvironment as well as showing high sensitivity to distinct stimuli originating from exogenous sources [146]. These distinctive characteristics have prevented the antibiotics’ premature release before arriving at the site of infection, thus improving the targeting of the bacteria and reducing adverse side effects and toxicities [147]. 

Moradi et al. have recently documented a pH-responsive polyvinyl alcohol/chitosan nanoemulsion that contains antibacterial thyme oil (TM). Results of the cytotoxicity testing of fibroblast cells have shown that 24 h post-contact, a percentage of 70–83% has been reported for metabolically active cells in the nanoemulsion as compared with cells lacking antibacterial agents, which suggests that the nano-emulsion can be a promising candidate to undergo in vivo assays [148]. Another study has investigated nanospheres as different nanocarriers for their potential relevance in antibacterial applications. In this study, pH-sensitive hybrid nanospheres have been prepared via a superficial molecularly imprinted polymer (MIP) technique combined with a method known as UV-initiated precipitation polymerization. Vancomycin (**33**) has been successfully embossed on a polymer made of 2-hydroxyethyl methacrylate, 2-(diethylamino) ethyl methacrylate, and ethylene glycol dimethacrylate which serves as a cross linker. Results of antibacterial evaluations have revealed that VA-MIPs show a greater antibacterial activity to *S. aureus*, as compared with NIPs, demonstrating that the prepared nanospheres can be promising for the management of bacterial infections [149]. Another study has prepared pH-responsive chitosan nanocapsules that have undergone polymerization with a phosphorylcholine-based copolymer. This system has reported an enhancement in the cell penetration as well as being able to localize the triclosan at the biofilm microenvironment of acidic nature. Results have also shown that these nanocapsules exhibit distinctive targeting properties which are employed to target the different bacterial surfaces, along with having a reduced adsorption level to *Staphylococcal* biofilm matrix, which resulted in an improvement in the eradication of *Staphylococcal* biofilms [150]. Niaz et al., in another study, designed pH-responsive nano-coacervates that were fabricated with cationic sodium caseinate and anionic sodium alginate. These nano-coacervates were loaded with nisin as the antimicrobial peptide [151]. Nanosheets are other systems of interest which, as reported in some studies, have shown a significant impact on the targeted antibiotic delivery mainly because of their high surface area and ability to functionalize their surface via ion exchange. Chahardahmasoumi et al. has developed pH-responsive montmorillonite nanosheets combined with tetracycline as the antibiotic. Despite the higher drug release show by the system, a simulation of the drug’s activity in a gastric media (pH = 1.2) was compared to the intestinal media (pH = 7.4) and showed its antibacterial efficacy against *E. coli* and *S. aureus* gave better results at pH 7.4. In addition, the system has also been shown to lose its antibacterial activity at a low pH (acidic) due to the formation of a complex with montmorillonite free ions, suggesting the potential of this system for effective antibiotic delivery in the intestine [145,152]. 

## 8. Antibiotic Adjuvants and Combination Therapies

**Figure 6:**
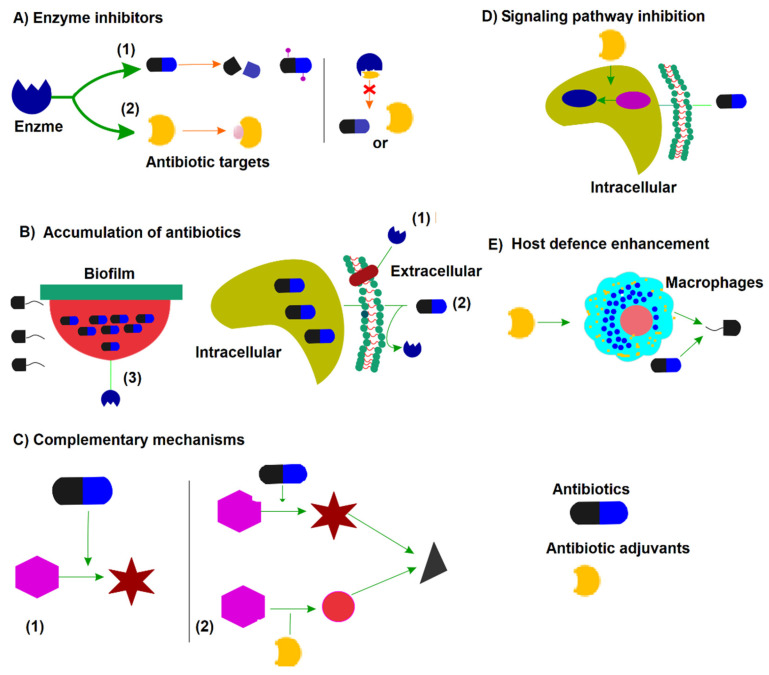
A schematic representation of the different mechanisms of action of antibiotic adjuvants. (**A**) Inhibition of hydrolase/modifying enzyme either on antibiotics as shown in (1) or the antibiotic targets as shown in (2); (**B**) enhancement of the intracellular accumulation of the antibiotic by the inhibition of efflux pumps as shown in (1), the facilitation of the antibiotic through the surface membrane as shown in (2) or the destruction of the biofilm as shown in (3); (**C**) the complementary mechanism; (**D**) inhibiting the signaling and regulatory pathway responsible for mediating the antibiotic resistance; (**E**) the enhancement of the host defense through the stimulation of the immune cells.

The use of antimicrobial adjuvants was shown to possess two main advantages against drug resistance as they increase the effectiveness of the antimicrobial and reduce the occurrence of the mutation, as shown in Figure 6 [39,41]. Combination of different antibiotics, such as trimethoprim-sulfamethoxazole or amoxicillin (**34**) with a β-lactamase inhibitor (for example clavulanic acid (**35**)), demonstrated higher efficacy in managing drug resistance. Currently, clinicians are combining two different classes of antibiotics, for example, a combination of β-lactams and aminoglycosides are used to achieve synergism when dealing with unknown pathogens or when suppressing the surfacing of drug resistance. Therefore, discovering adjuvant combinations of two molecules is believed to be an encouraging approach for reducing the incidence of resistance [52,62].

**Figure 7:**
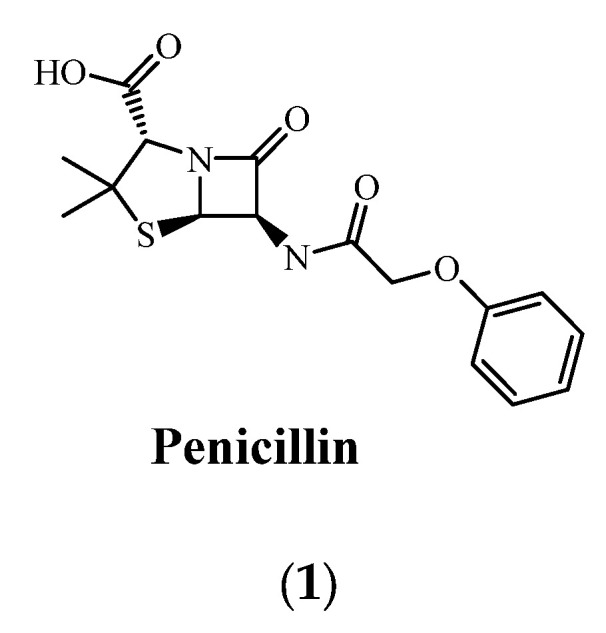

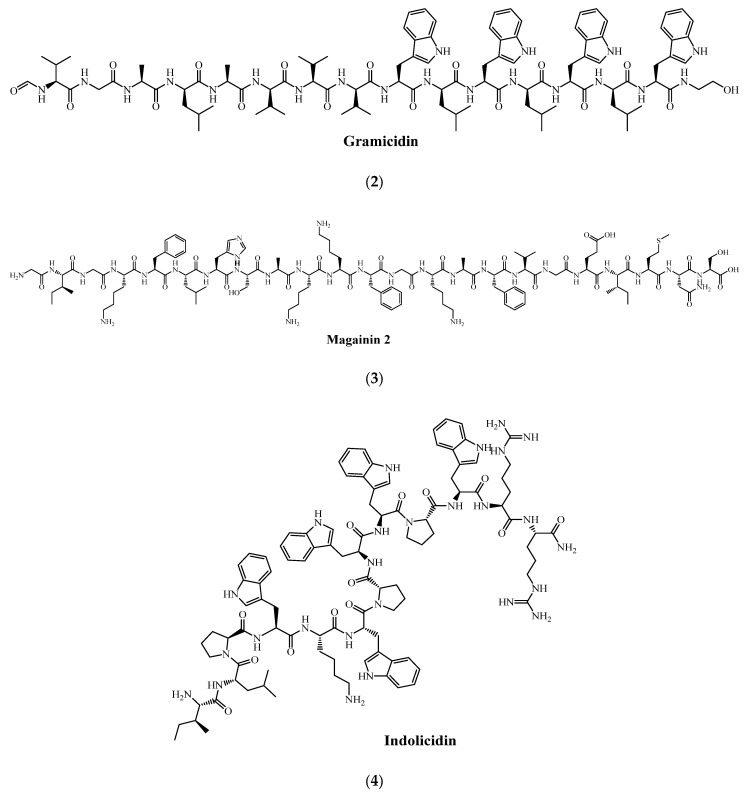

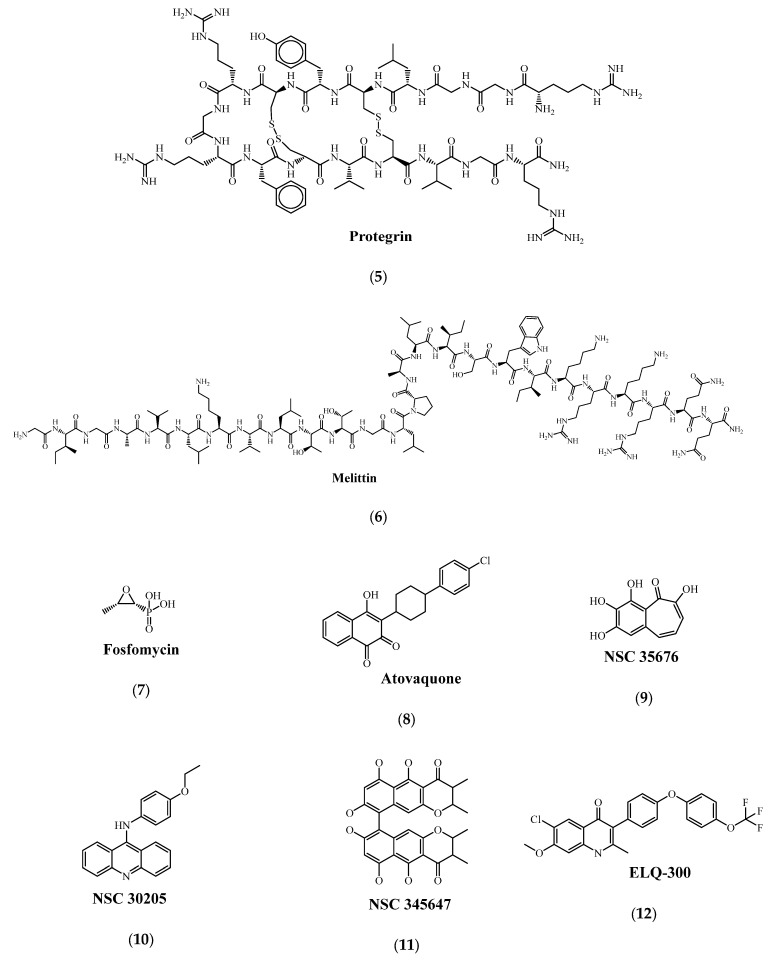

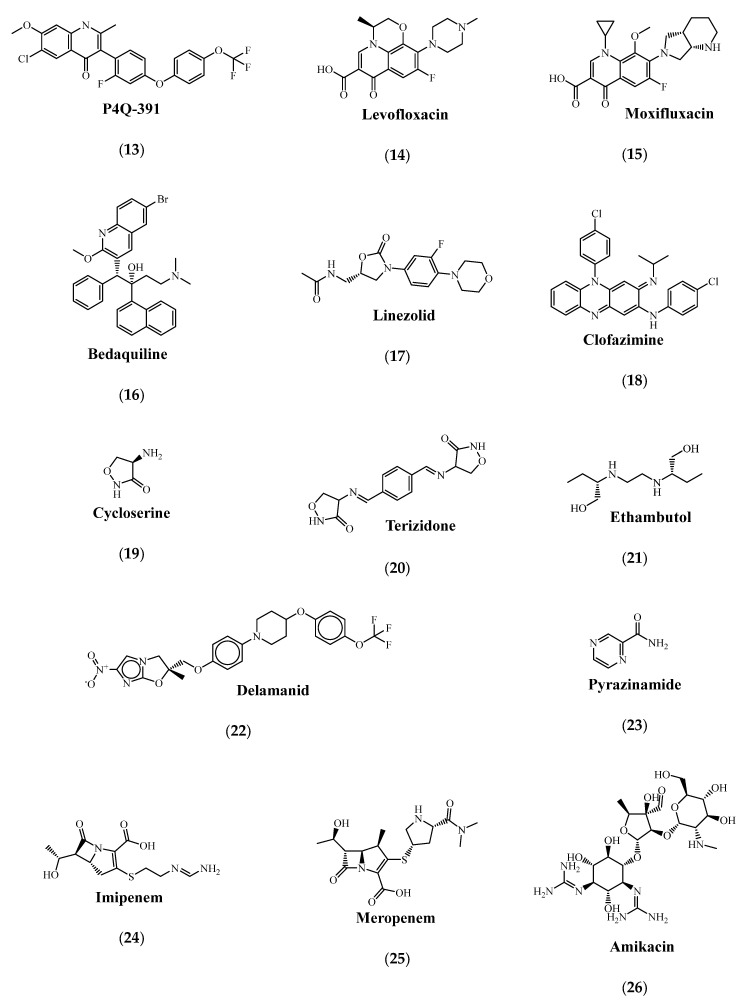

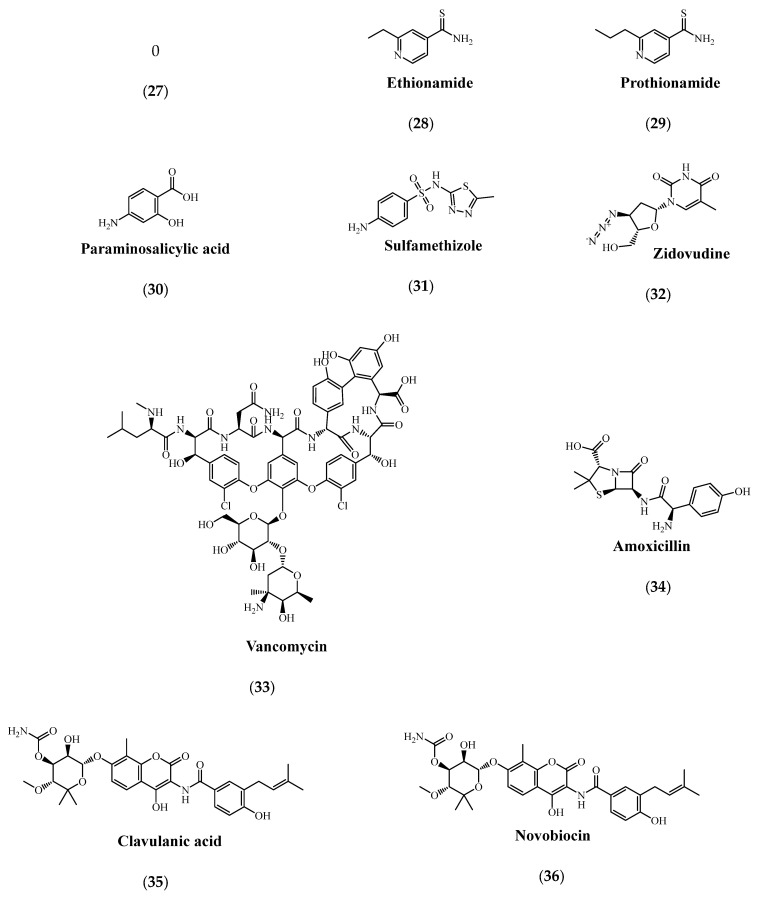

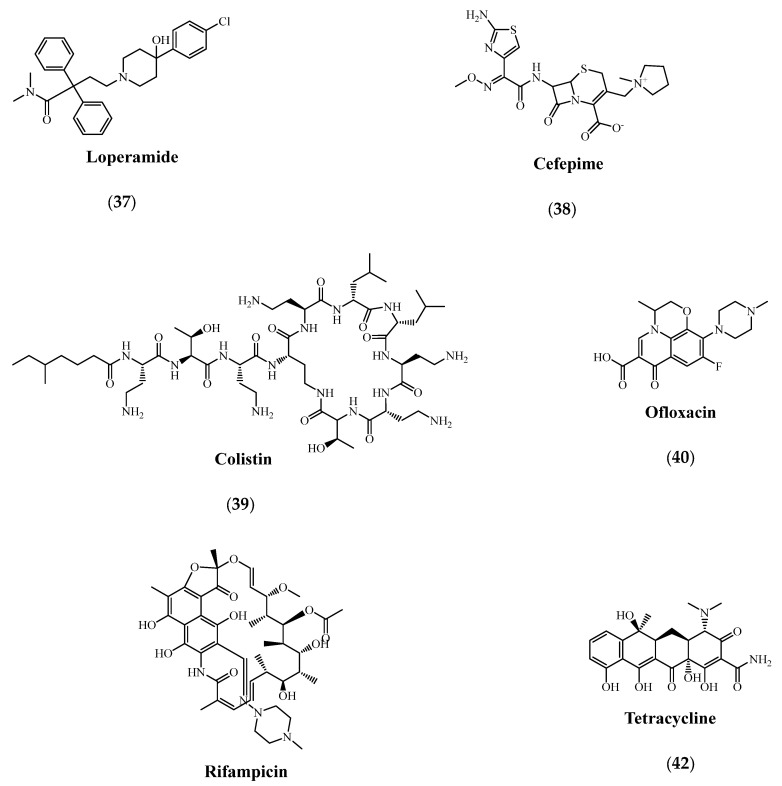
Chemical structures of antimicrobial agents.

In a study, the efficacy of an antibiotic, Novobiocin (**36**), in *E. coli* was improved using four novel compounds affecting the cell shape and membrane permeability. It has also been reported that loperamide (**37**) has been shown to induce potential membrane destabilization in bacteria, hence, increasing the minocycline’s permeability in gram-negative bacteria [153]. The new target-based discovery approach strives to identify the target selectivity with the least toxicity. Therefore, the adjuvant-mediated approach has an added advantage by bringing old antibiotics into use in this era of drug resistance. However, unexpected drug–drug interactions are an issue that has to be considered [41,154]. In recent research at the University of California, Los Angeles (UCLA), it has been stated that a combination of four to five antibiotics has been shown to stop or slow down the severity of infections caused by antibiotic-resistant bacteria. The researchers have applied a mathematical model—the mathematical analysis of general interactions of the components (MAGIC)—which has allowed them to predict the antibiotic combination’s result and enabled them to suggest 8000 theoretical combinations [155]. A recent animal-based experiment has demonstrated the effectiveness of bacterial toxin-grabbing nanoparticles, using machinery from bacteriophages that targets the antibiotic-resistant *Staphylococcus aureus* [41]. Since many bacteria are capable of evading the action of the antibiotic by limiting their access as well as accumulation in the cells, the use of membrane-active AMPs is an attractive approach for a synergistic effect with antibiotics. Zhu, Shen, et al. recently conducted a study where a short linear cationic AMP (SLAP)-S25 was reported to have the ability to restore the cefepime (**38**), colistin (**39**), ofloxacin (**40**), rifampicin (**41**), tetracycline (**42**) and vancomycin’s activity against MDR gram-negative pathogens [156]. AMP colistin combined with other antibiotics has been recently investigated and results have shown that this combination has resulted in a substantial decrease in the mortality rate of KPC infected patients as compared with other patients who received a monotherapy as a treatment [157,158]. Another strategy that attaches antibiotics to AMPs via a covalent bond that could, or could not, undergo cleavage has also been applied for developing antibiotic-AMP conjugates. Other scientists have applied this strategy to conjugate vancomycin to octaarginine via an amidation chemical reaction between the N-terminal amine and the carboxyl group of vancomycin [159]. Results have shown that the conjugate (V-r8) has effectively removed the biofilm formed by *S. aureus* and has reported a higher activity than vancomycin, octaarginine, or even the 1:1 mixture of vancomycin and octaarginine [160]. However, it is of great importance to note that these synergistic effects are variable. Some conjugates have been reported with a positive synergistic effect, whereas others have shown either no or a negative synergistic effect. Thus, to achieve the optimal synergistic effect, a careful exploration of factors such as linker cleavability and flexibility, modification sites, as well as conjugation chemistry is needed for a different case, as seen in Figure 7 [65].

## 9. Challenges and Future Outlook

Antimicrobial resistance forms a challenging barrier to the continual use of known and newly developed antimicrobial agents for the treatment of infectious diseases. Therefore, as antimicrobial resistance is becoming inevitable, it is necessary to continuously discover new drug candidates for better control of infections [161]. Moreover, investigating the antimicrobial resistance mechanisms can also be used as a guide and a research tool to discover new drugs [162]. For example, the discovery of successful chemical scaffolds including tetracyclines and aminoglycosides. A major stumbling block, the need for a full establishment of pharmacological and toxicological profiles, has been shown to contribute to the ongoing success of semisynthetic antibiotics that have both the pharmacological and toxicological profiles already established [11]. Moreover, lacking a rapid and reliable diagnostic has also exacerbated the prescription of inappropriate and/or unnecessary antibiotic combinations in clinics. Consequently, this has promoted the exposure of antibiotics and accelerated the emergence of resistance in the healthcare setting. In addition, the various genetic interactions have been exploited for different drug combination therapies to act against drug-resistant bacteria. Relying on the fact that the chemical-genetic signatures depend specifically on the species, the development of combinations of narrow-spectrum drugs can be accelerated [6]. The use of antibiotic adjuvants has been reported as an outstanding success: for example, the discovery of serine-β-lactamases inhibitors, which have been tested for the effective use in potentiating β-lactams activity as well as improve treatment outcomes in clinical trials [2]. Monitoring the impact of antibiotic resistance on the progress of some interventions is an important issue that has to be taken into consideration and which can be achieved by developing a specific indicator for AMR to meet sustainable development goals (SDGs) [163]. Most of the SDGs have given a clear definition of the performance indicators and the explicit actions needed to meet the set goals. Some have suggested that ensuring universal health coverage worldwide could broaden the visibility scope for sustainable development [164]. The assessment of the importance of having functional primary healthcare as well as the number of effective antibiotics available renders the inter-relatedness of SDGs and resistance more apparent [165]. These findings need to be addressed to government stakeholders to improve national agendas so that better actions can be considered against AMR [9]. 

## 10. Conclusions

The extensive use of antibiotics along with their misuse and overuse to treat different bacterial infections have accelerated the emergence of resistant microbial strains. The emergence of these resistant strains has undermined existing antimicrobial agents, which has therefore led to an inevitable global threat to public health. The various approaches discussed in this review are currently under investigation, undergoing clinical trials or available commercially. A recent report from the WHO in 2019 revealed that AMR to tuberculosis drugs accounts for about 1.6 million annual deaths in 10 million cases. Another report published by the CDC in 2019, showed that about 3 million antibiotic-resistant infections occur annually in the USA, leading to about 35,000 deaths. Consequently, the identification of new effective therapeutic strategies has become urgent. However, the continuous increase in the rates of failure to discover new antibiotics that meet the efficacy and safety requirements is quite challenging, thus interest in using combined therapies among those in the drug discovery field is growing. The GLASS report, published last year, has shown a significantly increased resistance in common infections to the available antibiotics. Recently, various health institutions worldwide have raised awareness toward the misuse of antibiotics, especially during the COVID-19 pandemic. As a result, researchers have recently begun to increase efforts to develop alternative technologies that would circumvent this global crisis. A significant escalation in the design and development of advanced nanomaterials has been witnessed globally. Intrinsic stimuli-responsive antibiotic nano carriers have been recently introduced as an alternative due to their improved activity and targeted delivery. Carbon nanodots, composed of multiple layers of oxidized graphene sheets are also a promising alternative.

Global antibiotic resistance is not showing any signs of decline. According to a WHO estimation, AMR is anticipated to be the leading cause of death for 10 million people every year by 2050. Despite massive efforts to develop new antimicrobial agents, a principally coordinated political campaign is still missing worldwide. Regulations need to be applied with a high level of restriction to monitor the use of antibiotics as a part of their policy. Consideration of global and interdisciplinary approaches is a must to develop new tools for screening and diagnosis. Nonetheless, other aspects governing the effect of antibiotic misuse of the ecology and environment must not be ignored.

## Figures and Tables

**Table 1:** The various timeline-dependent strategies implemented for antimicrobial drug discovery.

Era	Year(s)	Approach
Golden era	1940–1962	Research-based on natural products Identification based on Whole-Cell screening
The era of Medicinal chemistry	1950–1980	Synthetic tweaking Identification based on Whole-Cell screening Compounds showing broad-spectrum activity
The era of antimicrobial Resistance	1960 onwards	Modern methods of drug discovery Target-based drug design Ligand-based drug design Compounds showing broad-spectrum activity
Era of narrow-spectrum	2025	Unconventional methods for drug design and discovery Combinatorial synthesis Diagnostic development

**Table 2:** Current availability of antimicrobial peptides (AMPs) databases [81].

Database	Description
Collection of antimicrobial peptides (CAMP)	Holding experimentally validated and predicted AMP sequences
AMPer	Database and automated discovery tool that is used for gene-coded AMPs
Antimicrobial Peptide Database (APD)	Containing the AMPs from natural sources (~98%)
BACTIBASE	Data repository of bacteriocin AMPs
PhytAMP	Database of plant base antimicrobial peptides
RAPD	Database of AMPs produced by recombinant technology
HIPdb	Peptides showing anti-HIV activity
Bagel2	A tool for bacteriocin mining
Peptaibol	Database for peptaibols
PenBase	Database for penaeidins
Defensins Knowledge Base	Database for defensins
CyBase	Database for cyclotides

**Table 3:** The major target genes that confer resistance in the treatment of tuberculosis in cases of mutations.

Drug Group	Drug	Target Gene	Reference
A	Levofloxacin (**14**) or Moxifluxacin (**15**)	*gyrA*	[114]
	Bedaquiline (**16**)	*atpE*	[115,116]
	Linezolid (**17**)	*rplC*	[117]
B	Clofazimine (**18**)	*rv0678* *rv1979c*	[118]
	Cycloserine (**19**) or Terizidone (**20**)	*Rv2535c* *alr*	[119,120]
C	Ethambutol (**21**)	*embCAB*	[121,122]
	Delamanid (**22**)	*ddn* *fgd-1* *fbiA* *fbiB* *fbiC*	[123]
	Pyrazinamide (**23**)	*pncA* *rpsA* *panD* *clpc1*	[124]
	Imipenem (**24**)/Clavulanic acid (**35**) or Meropenem (**25**)	*rv2518c* *rv3682* *rv2068c*	[125]
	Amikacin (**26**)	*Rrs*	[126]
	Streptomycin (**27**)	*Psl*	[127]
	Ethionamide (**28**) or Prothionamide (**29**)	*Rv0565c* *ethA* *mymA* *katG* *inhA*	[128]
	Paraminosalicylic acid (**30**)	*thyA* *folC* *dfrA*	[129]

## Data Availability

Data sharing is not applicable.

## Abbreviations

AMP	Antimicrobial peptide
AMR	Antimicrobial resistance
anhMurNAc	1,6-anhydroumuropeptides
ARGs	Antibiotic resistance genes
ARP	Antibiotic resistance platform
ATP	Adenosine triphosphate
BrCND	Brominated carbon nanodot
CDC	Center for disease control and prevention
CRE	*Carbapenem-resistant Enterobacterales*
ESKAPE	*Enterococcus faecium, Staphylococcus aureus, Klebsiella pneumoniae, Acinetobacter baumannii, Pseudomonas aeruginosa*, and *Enterobacter species*
FA	Fatty acid
FDA	Food and drug administration
GLASS	Global antimicrobial resistance and surveillance
GlcNAc	N-acetylglucosaminyl
GSK	GalaxoSmithKline
MD	Molecular dynamic
MDR	Multi-drug resistance
MIP	Molecularly imprinted polymer
MM-GBSA	Molecular-generalized born surface area
MMV	Medicine for malaria venture
MRSA	Methicillin-resistant *Staphylococcus aureus*
MTB	Multi-drug resistant tuberculosis
NIPs	Non-molecularly imprinted polymers
O2M	Overlap2 method
PG	Peptidoglycans
PolyP	Polyphosphates
PPK	Polyphosphate kinase
QSAR	Quantitative structure-activity relationship
SAR	Structure-activity relationship
SDGs	Sustainable development goals
UV	Ultraviolet
VRSA	Vancomycin-resistant staphylococcus *aureus*
WHO	World health organization
XDR	Extensively drug-resistant
